# The Buzz Surrounding Precision Medicine: The Imperative of Incorporating It into Evidence-Based Medical Practice

**DOI:** 10.3390/jpm14010053

**Published:** 2023-12-29

**Authors:** Guido Muharremi, Renald Meçani, Taulant Muka

**Affiliations:** 1 Epistudia, 3008 Bern, Switzerland; guido.muharremi@epistudia.com (G.M.); renald.mecani@epistudia.com (R.M.); 2 Division of Endocrinology and Diabetology, Department of Internal Medicine, Medical University of Graz, 8010 Graz, Austria

**Keywords:** evidence-based medicine, precision medicine, interdisciplinary approach, clinical medicine, public health, meta-analysis

## Abstract

Precision medicine (PM), through the integration of omics and environmental data, aims to provide a more precise prevention, diagnosis, and treatment of disease. Currently, PM is one of the emerging approaches in modern healthcare and public health, with wide implications for health care delivery, public health policy making formulation, and entrepreneurial endeavors. In spite of its growing popularity and the buzz surrounding it, PM is still in its nascent phase, facing considerable challenges that need to be addressed and resolved for it to attain the acclaim for which it strives. In this article, we discuss some of the current methodological pitfalls of PM, including the use of big data, and provide a perspective on how these challenges can be overcome by bringing PM closer to evidence-based medicine (EBM). Furthermore, to maximize the potential of PM, we present real-world illustrations of how EBM principles can be integrated into a PM approach.

## 1. Introduction: The Growing Field of Precision Medicine

Precision medicine, often referred to as personalized medicine, is currently one the most prominent approaches in modern healthcare and public health. The phrase “Personalized medicine” has been commonly utilized in the past. However, because it was often misinterpreted to imply treatments catering only to a particular individual, it has been replaced worldwide by the term “precision medicine” (PM) [1]. Precision medicine does not create custom drugs or unique medical devices for individual patients. Instead, it aids in categorizing individuals and subpopulations based on their susceptibility to certain diseases or their response to a particular treatment.

Unlike the conventional approach, PM departs from the one-size-fits-all approach by integrating information on individuals’ omics (genetics, epigenetics, proteomics) and environmental factors (including lifestyle) to provide a more precise prevention, diagnosis, and treatment of disease [2]. Due to the huge size and complexity of omics data and the dataset of patient features required for PM, they cannot be analyzed directly by doctors. Big data is a term used for complex or large datasets that cannot be accurately processed or stored by traditional management tools. Omics and electronic health record (EHR) data are essential big biomedical data, forming the foundation of PM [3].

The phrase “precision medicine” has gained significant popularity in recent years, driven not only by scientific community, but also from entrepreneurial and political agendas, most notably with President Obama addressing and launching a new Precision Medicine Initiative in his 2015 State of the Union address as *“a bold new research effort to revolutionize how we improve health and treat disease”* [4]. There is no doubt that the increased exposure of science, and in this case PM, to the general public is a positive development, as it provides people with a greater understanding of scientific principles, and furthermore involves them as more active members in matters related to healthcare and healthcare policy.

**Figure 1:**
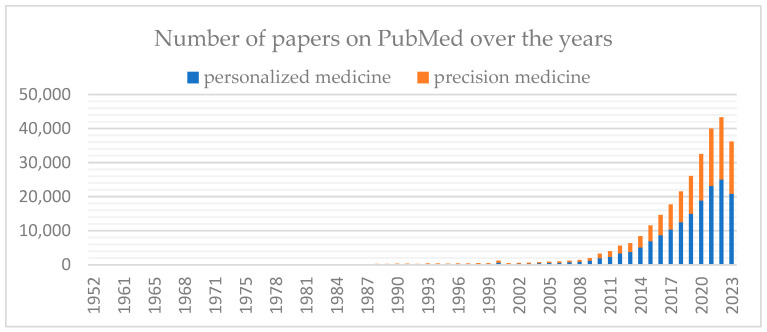
The number of papers on ‘‘precision medicine’’ and “personalized medicine” in the PubMed database worldwide. Articles until September 2023.

The rising popularity of this trend is also evident through the growing number of research papers on “precision medicine” and “personalized medicine”, reaching more than 40,000 publications yearly in the PubMed database (Figure 1).

**Figure 2:**
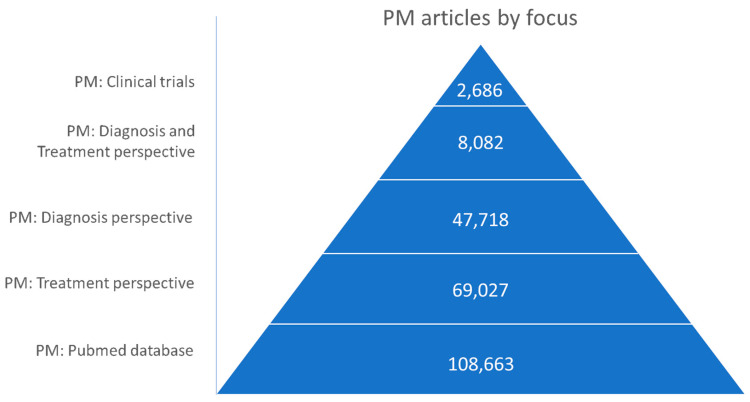
The number of papers on ‘‘precision medicine’’ and “personalized medicine” in the PubMed database, categorized into subsets based on their specific focal point.

The focus of the research papers dealing with “precision medicine” and “personalized medicine” is predominantly on the treatment aspects of PM, with an emphasis on drug development. This focus is closely followed by the diagnosis perspective in the research landscape. Only a fraction of the articles deals with both treatment and diagnostic aspects, while an even smaller proportion examines the clinical trial landscape of PM (Figure 2).

**Figure 3:**
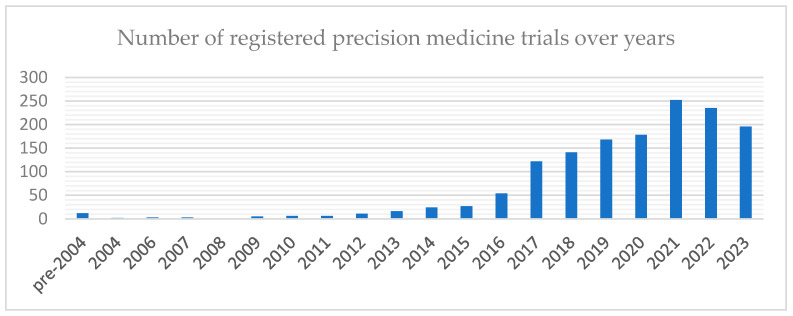
The number of registered precision medicine trials per year in the clinicaltrials.gov database worldwide. Trials registered until December 2023.

The popularity of precision medicine is also reflected in the increasing number of clinical trials that incorporate it. The clinicaltrials.gov database currently contains 1938 clinical trials involving PM, with an increasing trend over time (Figure 3).

In this article, given the widespread interest in precision medicine, we provide a critical overview of the current challenges facing precision medicine. We also present a perspective on addressing these challenges by aligning precision medicine more closely with evidence-based medicine (EBM).

## 2. Precision Medicine: Exaggerated or Promising?

Despite its increasing popularity in scientific circles over the past few years, PM still faces considerable challenges that need to be addressed and resolved for it to attain the acclaim for which it strives. One of the most significant challenges that persistently hinders the adoption of PM and, more importantly, the potential for further research in this field, pertains to the generation and management of “big data”. The main issues found in a study by Di Sanzo et al. [5] include the typical storage of data in a different way in different hospitals and the fact that the EHRs do not support formats needed to record genetic results. These issues place extreme limits on the standardization of data, their study and interpretation, and then their use. According to a study completed by Schneuer et al. [6], among the clinicians participating in the study, few felt that their EHR met their genetic/genomic medicine needs, mostly because they perceive genetics to be a low priority of EHR.

Another problem when dealing with EHR and big data in the context of PM is analytical capacity. Artificial intelligence (AI) is becoming increasingly popular in industries like healthcare to manage and analyze EHR and big data. Thanks to AI, it is possible to analyze large datasets and to extract calibrated intervention models that would otherwise be impossible; for example, the identification of more accurate prognostic, diagnostic, and predictive markers for specific diseases [7]. Despite the tremendous progress that has been made in predicting outcomes through the use of AI techniques and genomics, there are still some challenges in harnessing the power of AI, including those related to the pitfalls of using EHRs, such as the presence of inherent biases [8]. Most data documented in EHRs are influenced by the indications for treatment and policies, which encompass insurance, both at the level of hospitals and within nations, thus intruding bias at different levels that are difficult to address when analyzing the data, challenging the interpretation of results. An article by Verheij et al. [9] summarized various sources of bias in EHR data within primary care settings, such as variations between EHR system functionalities and lay-out, or health care system bias, emanating from the role of the general practitioner in the health care system; gatekeeping/non-gatekeeping, the professional clinical guidelines, and data sharing between health care providers. All these factors could also contribute to the paradoxical phenomenon of “big data”, which encompasses a real-world phenomenon whereby as the number of patients enrolled in a study increases, the probability that the confidence intervals from that study will include the truth decreases [10]. Therefore, even though PM relies on extensive data, larger data volume does not necessarily equate to better quality, less false findings, and better health outcomes for population and patients [11].

Another drawback to PM is its relative novelty in the field, as stated in the beginning. The recognition of precision medicine as a paradigm suitable for widespread acceptance within the biomedical research and clinical communities is a relatively recent development. Therefore, insufficient time has passed since this acknowledgment for researchers to demonstrate the efficacy of personalized medicine across a diverse range of scenarios, thereby encouraging its extensive clinical adoption [12]. This could potentially pose an issue. For example, numerous startups in the lifestyle and digital health sectors, also driven by patients considering personalized medicine as the standard of care [13], have founded their entire enterprise on PM principles. These start-ups use data from EHRs and smart devices such as smart watches and continuous glucose monitors. This is due to the ease and cost-effectiveness of gathering, processing, and analyzing these data, enabling the creation of personalized diagnostics and treatment plans. However, many of these digital health companies have low levels of clinical robustness to support their claims, indicating a need for further clinical validation [14].

## 3. Aligning Precision Medicine with Evidence-Based Medicine: Is This the Future? 

Evidence-based medicine provides a framework for applying the relevant scientific evidence to the patient’s condition by integrating the level of evidence, patient’s values, and clinician’s clinical judgment to tailor the treatment for the patient [15]. EBM principles have also been expanded in tailoring recommendations to the population level, primarily focusing on preventative measures. EBM utilizes findings from all study designs in a hierarchical level, with findings from high quality meta-analyses [16,17] and randomized control trials (RCTs) weighing the most in formulating and establishing general and specific clinical and public health recommendations [18]. 

The integration of EBM principles into PM could hinge on predictions based on a mechanistic knowledge of genetic or environmental influences on drug responses. These insights can be empirically examined and integrated into the planning of RCTs. An example to maximize and tailor the clinical benefits of certain drugs would be to stratify patients by genetic markers according to our knowledge of pharmacogenetics. The CYP2D6 gene variation is the best understood; one extreme is the poor metabolizer who does not have any CYP2D6 due to a lack of functional genes and the other extreme is the ultrarapid metabolizer who has too much CYP2D6 enzyme due to three or more active genes. Ideally, RCTs stratified by CYP2D6 genotype should be carried out for classical antidepressant and antipsychotic drugs that are off-patent [19]. An increasing number of studies have followed this rationale, sub-stratifying patients and linking pharmacogenetic (PGx) variability of CYP2D6 and CYP2C19 to drug blood-concentrations, the treatment response, and the remission rates in patients with depression [20], aiding in the creation of the Clinical Pharmacogenetics Implementation Consortium (CPIC) Guidelines for CYP2D6 and CYP2C19 Genotypes [21]. 

Building upon this rationale, refining EBM guidelines for enhanced compatibility with PM could mark a potential advancement in methodological approaches, aiming to further optimize the potential of precision medicine. An example of such an approach is seen in “N-of-1 trials.” In cancer studies, though these trials include multiple participants, each patient is treated individually, based on the principles of tumor biology. The allocation strategy in these trials stands apart from traditional methods. In the former, therapy is tailored to the individual, whereas in the latter, the patient is adapted to the drug. Similar applications of the “N-of-1” methodology can be useful in behavior-modification studies [22].

Another example, based on behavior-modification studies on neurobehavioral disorders, is the precision clinical trial (PCT) proposed by Lenze et al. [23]. This framework comprises a set of clinical trial design decisions aimed at delivering optimized interventions to individual patients who are most likely to benefit. The key point is that the PCT is a pragmatic framework that acknowledges that neurobehavioral disorders are complex, and their pathophysiology is largely unknown, so that the path to optimized treatments for individual patients requires different clinical trial methods than standard RCTs [23].

PM has been disproportionately focused on drug development and strategies for those who have a disease with an intention to improve outcomes, leaving behind the concerns of whole population health [24]. Incorporating EBM principles into the scope of PM, through discovering pertinent research studies, rigorously evaluating their quality, and seamlessly incorporating the evidence into clinical and public health practices could help shift the PM paradigm from drug development to prevention and becoming a powerful tool of public health. One positive example would be the use of risk stratification models to identify patients that are at a higher risk of COVID-19 infection and severe illness [25]. These models have made possible the prioritization of patients requiring closer follow-up by their physicians and outreach services, as well as identifying those that are most likely to benefit from anti-viral treatment during the fifth wave of infection in Israel, dominated by the Omicron variant [26]. Precision public health by using more accurate measures of disease spread, susceptibility, and behavior to assess population health and develop targeted programs can also support broader COVID-19-related public health efforts [27]. 

In the realm of precision preventive medicine, an ongoing area of exploration involves examining how particular dietary factors or dietary patterns may influence the genetic susceptibility to cardiovascular diseases. Although there have been significant enthusiasm and substantial investments in this area, a recent review indicates that the available evidence regarding gene–diet interactions in cardiovascular diseases is quite limited, suffering from many methodological limitations [28]. 

## 4. Outlook for Integrating Evidence-Based Medicine into Precision Medicine

The establishment of a framework that combines precision medicine and evidence-based medicine requires various initiatives, such as the implementation of protocols, addressing ethical concerns, incorporating GRADE (Grading of Recommendations, Assessment, Development, and Evaluations) approach, and evaluating the cost-effectiveness of personalized medical interventions, as well as designing educational programs focused on PM.

(a) Implementation of Protocols

A comprehensive research protocol umbrella streamlines the feasibility testing of operational workflows, the early collection of internal data not only to preserve quality and limit inherent biases but also to assess utility, and the development of a portfolio for precision medicine trials. This approach may require synchronized efforts in protocol development, tissue procurement, sample transportation, molecular testing result analysis, and the functioning of a molecular board to ensure timely patient care and enrollment in precision medicine trials. Managing the intricacies of a precision medicine program is best handled by a multidisciplinary team led by a dedicated leadership [29]. The establishment of specific clinical protocols, incorporating a precision medicine approach in community-based oncology practices conducting clinical trials where the majority of cancer patients (85%) receive treatment, offers a significant opportunity to impact precision medicine trial accrual through the successful operationalization of a community-based precision medicine program [30]. Within this context, well-designed feasibility studies are needed to test whether PM can be beneficial. One such example is a pilot project [31] carried out by three clinical facilities in the USA. The goal of the project was to determine whether a precision medicine approach to treating Alzheimer’s disease and mild cognitive impairment was effective enough in a proof-of-concept study to warrant a larger, randomized, controlled clinical trial. Despite some limitations of the study (no direct comparison with pharmacologically treated or untreated patients; inadequately representative of a racially diverse group, as the patients who responded to the study announcement and became study participants were predominantly white), the results of this proof-of-concept trial, based on the cognitive improvements observed in this study, warranted a larger, randomized, controlled trial of the precision medicine therapeutic approach.

(b) Ethical considerations

Another aspect to address involves the numerous ethical challenges associated with PM [32]. One important ethical issue is addressing patients’ primary concerns of privacy, confidentiality, and security [33]. Within this context, as genetic testing plays a crucial role in personalized medicine, twin studies, particularly those involving identical twins, are a good example of such ethical issues. Privacy issues arise when one twin undergoes genetic testing, and the results may indirectly reveal information about the other twin without their explicit consent. This situation poses challenges affecting the untested twin’s privacy, autonomy, and well-being, and raises concerns about autonomy and control over genetic information, as the scenario risks exposing one twin’s data without consent, prompting ethical questions. Respecting both twins’ autonomy and privacy is crucial. Therefore, conducting genetic testing or sharing data without explicit consent from both twins requires careful ethical consideration to safeguard individual autonomy and privacy. A possible approach when dealing with such ethical issues is suggested by Vayena and Gasser [34], proposing three main strategies differing from the traditional approach: (a) expanded safeguards—covering all stages of data handling in research and sharing, moving beyond just data collection consent; (b) advanced tools integration—incorporating innovative computational tools like differential privacy and data tagging systems to manage privacy risks effectively; (c) systemic awareness—emphasizing the interplay between privacy, openness, and their potential harms or benefits at a broader level.

Another ethical issue concerns implementation, i.e., establishing the threshold for a sufficient level of certainty in the evidence to justify adoption and implementation in clinical practice and public health. Factors such as the quality of available evidence, knowledge of potential harms, and the benefit-to-harm ratio are among those that should be discussed in the development of clinical practice guidelines and the implementation of PM. For trustworthy clinical practice guidelines, it is also important to consider conflicts of interest. A recent study has shown that the most influential clinical trials are sponsored by industry, which affects not only funding but also authorship and a positive outcome [35]. Obviously, this is not an issue exclusive to PM, as the majority of RCTs are also funded or co-funded by industry [36]. Therefore, maintaining the integrity and ethics of future trials is an issue to be dealt by both PM and EBM approaches.

Another important ethical issue is the question of what is suitable for the implementation of personalized medical interventions, considering the available evidence and uncertainties. For example, when a personalized test is introduced into clinical practice, clinicians and patients need to discuss the uncertainties with which they are most comfortable and what factors would limit or support the use of a new precision medicine intervention. In addition, the likely higher costs of precision medicine technologies, big data management, and health policy pose the risk of unequal access to precision medicine. Certainly, unequal access is a concern within EBM as well, but in the case of PM could particularly affect marginalized populations who may not have access to genetic testing or targeted therapies, which could widen the gap between less economically developed countries and more economically developed countries. In this case, the question arises as to who will bear the costs. A European study indicates that health insurers are hesitant to cover the expenses for PM due to inadequate evidence and insufficient incentives [37]. A collaborative effort from multiple stakeholders, including governments and pharmaceutical companies, as suggested by Lu et al. [38], seems like a promising direction. The authors proposed an innovative model where governments and industries collaborate to share both the risks and rewards of drug development. They advocate for clinical trials becoming a standard part of healthcare, foreseeing improved healthcare outcomes alongside potential economic advantages. Obviously, the implementation of such a proposal would require careful structuring. Collaborative efforts between governments and industry could potentially lead to a fairer distribution of the costs and benefits of drug development. Governments can ensure that these developments are accessible and affordable to those in need. Simultaneously, by sharing risks and benefits, it could incentivize pharmaceutical companies to invest in research for medications that might not have a huge market but could significantly benefit certain populations. This is also a concern among patients that if a medication is not prescribed frequently or falls into a specific category that is destined to treat rare diseases, pharmaceutical companies might increase its price or discontinue its production [39]. Rare diseases are an important area in which PM holds a lot of promise, as showcased in an article by Might and Crouse [40]. The authors argued that PM is easier for rare diseases because the root molecular cause becomes apparent almost immediately with the diagnosis itself, which is exactly the type of root cause that PM aims to identify in every patient, rare or common. In addition, the systematic study of patients suffering from rare diseases can contribute to the identification of therapies for patients suffering from complex and multifactorial diseases, ultimately resulting in the “collaboration” between PM and EBM.

(c) Cost-effectiveness and translating evidence into clinical and public health recommendations

By tailoring medical approaches to individuals based on their genetic makeup, lifestyle, and environment, PM aims to enhance treatment efficacy and reduce adverse effects. Adverse drug reactions’ (ADRs) clinical costs in patients can be translated to economic costs as well, as they often lead to hospital admissions, emergency department visits, and so forth. While being a fragile point for RCT and EBM practices [41], reduction in ADRs, through pre-emptive pharmacogenetic testing using a pharmacogenetic panel [42], might translate into fewer economic costs in the future. Therefore, advocates of PM point out that it has the potential to be cost-effective in the long term. In some cases, the upfront costs of PM may be higher due to personalized diagnostics or treatments, but it can potentially lead to long-term cost savings by avoiding ineffective treatments or reducing complications. Also, the popularization and its increased application in the future might be followed by rapidly decreasing costs, just as happened with DNA sequencing from USD ~1-3billion in 2001–2003 to USD 13–30 thousands [43,44]. 

Henderson, R.H. et al.’s [45] study underlined the potential cost-saving benefits of incorporating companion diagnostics (CDx) in early oncology medicine development. By integrating CDx, there could be a significant reduction in developmental costs, offering a pathway to enhance accessibility within constrained cancer health systems. Chen W. et al. [46] argued that this development in PM demands a reassessment of existing guidelines or the creation of new reference frameworks to effectively navigate the evolving healthcare landscape. The integration of PM introduces complexities beyond mere cost reduction, urging a comprehensive review of decision-making frameworks.

Yet, amidst these promising strides, Vellekoop et al.’s [47] systematic literature review brings attention to the challenge of cost-effectiveness in PM. Their analysis reveals that while PM brings health improvements, it often does so at a considerable cost, sometimes resulting in neutral or negative net monetary benefit (ΔNMB). Pricing policies might be necessary to mitigate the cost of interventions displaying negative ΔNMB, as highlighted in their findings. Kasztura et al.’s [48] scoping review emphasized the multitude of variables influencing the cost-effectiveness of PM, coupled with varying “willingness-to-pay” thresholds. This variability makes it challenging to conclusively ascertain the cost-effectiveness of PM in practical terms. 

Therefore, addressing the complexities of cost-effectiveness, guideline adaptations, and diverse thresholds of acceptance are pivotal in harnessing the full potential of PM. It is about striking a balance between cost-efficiency and ensuring that decision-making processes in healthcare encompass the multifaceted aspects introduced by the era of PM.

Employing the GRADE approach can aid in transforming existing evidence into evidence-based recommendations. However, additional efforts may be necessary to tailor GRADE to the specifications of PM. For example, applying GRADE to genetic studies has revealed certain limitations that need to be addressed [49]. Subbiah V. et al. [50] proposed the idea of an “evidence-based deep medicine iceberg”. EBM pyramids are described by the author as the tip of an iceberg, whose foundation underwater is made up of a massive amount of data, which consist of a combination of genomic, proteomic, metabolomic, microbiome, and imaging data, PRO-based data, as well as real-world data, and that by amalgamating all these data will facilitate PM—that is, to offer the correct treatment at the appropriate time to the right patient. 

Therefore, applying GRADE to PM requires a multidisciplinary and personalized approach addressing different study designs, approaches, disciplines, and diverse amount of data. For instance, most PM clinical trials are based on a small sample size; thus, the meta-analysis required for GRADE may require a different approach from EBM at translating the evidence into recommendations. For example, stratification employed in meta-analysis of clinical trials, including meta-analysis on individual patient data (IPD), has the potential to offer novel perspectives on targeted approaches in the context of PM applications [16,51]. Thus, replicating the outcomes derived from these meta-analyses in a subsequent clinical trial could establish a level of evidence not anticipated in the traditional EMB approach.

(d) Designing educational programs

Lastly, preparing all healthcare and public health providers for the transition to a PM–EBM era is also crucial. While PM is emerging as a new direction shaping the future of healthcare, there is a lack of adequate training and a new specialty or curricula focused on PM is still lacking. For example, the rise in data accessibility has coincided with a deficit in individuals equipped to analyze and interpret those data [52], leading in turn to a higher need of translational research experts to combine and integrate these data types. The inability of doctors to effectively analyze big data should not pose a problem to PM’s implementation, as equipping with integrated data could be a viable way forward for both EBM and PM. At the same time, “the personalization” of EBM practices, evidenced by the increasing number of drugs to have received pharmacogenetic drug labels from the FDA or EMA, is a sign that pharmacogenomics testing has significantly contributed to improved EBM delivery through PM. However, a challenge remains to expand the application of pharmacogenomics from oncology to other specialties as well, as cancer has received the most FDA drug approvals with pharmacogenomics labeling from 2000 to 2020 [53].

This could be achieved by expanding the educational offerings in medical education to all healthcare professionals—physicians, nurses, pharmacists, and other staff—by highly skilled experts involved in PM in clinical practice. Such an approach can facilitate the implementation and quality assurance of PM across all disciplines, and may do so. Likewise, in the future, we might amplify the enhancement by delivering the content through conventional continuing pharmacy education programs, which can be offered within national meetings or as independent programs [54].

## 5. Conclusions

Despite these challenges, PM continues to hold promise in improving healthcare outcomes, particularly in areas like cancer treatment, rare disease diagnosis, and drug development. Overcoming these issues will require collaboration, technological advancements and continued research and development efforts. The co-existence of PM and EBM is a crucial feature to the future of unlocking PM’s full potential, and assessing PM for its effectiveness, cost-effectiveness, and safety. In addition, it is necessary to redefine EBM’s guidelines to align them with PM, in the form of “N-of-1” trials or precision clinical trials, as mentioned above, prior to its widespread adoption. By addressing the need for the future training of medical practitioners, broadening the scope of training within medical education programs to encompass all healthcare professionals as adept specialists involved in personalized healthcare within clinical practice, can significantly aid in reaching this objective.

## Data Availability

Not applicable.
